# Association between estimated glucose disposal rate and nephrolithiasis: a propensity score matching study in US adults: results from the National Health and Nutrition Examination Survey 2011–2020

**DOI:** 10.1080/0886022X.2026.2684346

**Published:** 2026-07-28

**Authors:** Chengxin Li, Yurun Xie, Weizhou Pan, Deqiang Qin, Hua Mi

**Affiliations:** ^a^ Department of Urology, The First Affiliated Hospital of Guangxi Medical University, Nanning, Guangxi, China; ^b^ The Key Laboratory of Clinical Diagnosis and Treatment Research of High Incidence Diseases in Guangxi, Department of Urology, The Affiliated Hospital of Youjiang Medical University for Nationalities, Baise, Guangxi, China

**Keywords:** Insulin resistance, nephrolithiasis, estimated glucose disposal rate, national health and nutrition examination survey, relationship

## Abstract

**Background:**

The estimated glucose disposal rate (eGDR) is a reliable substitute for insulin resistance markers. However, a thorough assessment targeting the relationship between eGDR and nephrolithiasis (NL) occurrence has not been performed.

**Methods:**

In total, 31,566 participants from the National Health and Nutrition Examination Survey 2011–2020 were enrolled in our analysis. We performed the weighted multivariable logistic regression to evaluate the correlation between the eGDR and NL history. Restricted cubic spline curves were used to further explore this correlation. We also conducted similar statistical analyses in the population after a 1:1 propensity score matching (PSM). Receiver operating characteristic (ROC) curves were utilized to determine the predictive power of eGDR for the NL history.

**Results:**

Our analyses revealed a negative relationship between eGDR and the NL history. Subgroup analysis demonstrated that the association was consistent with the significant outcomes in different stratifications. The analysis after PSM still demonstrated that the eGDR level was negatively related to NL history. In ROC analysis, eGDR had a more considerable value of area under the curve (AUC) compared to visceral adiposity index (VAI), triglyceride-glucose (TyG) index, and body mass index (BMI). The AUC values were 0.62 for eGDR, 0.56 for BMI, 0.52 for TyG, and 0.53 for VAI, with all pairwise comparison *P*-values < 0.001.

**Conclusions:**

The eGDR was negatively associated with NL history among the United States population aged ≥ 20. Moreover, eGDR had greater predictive power for NL history than VAI, TyG, and BMI.

## Introduction

Nephrolithiasis (NL)—as one of the most common urological diseases in the world—can result in severe complications, such as kidney damage, local or systemic infection, and even renal failure and septic shock [1]. According to estimates reported by Sui, Wilson et al. worldwide NL prevalence has reached 2%–15% [2]. A dramatic escalation in NL incidence has been observed over the past three decades [3]. Moreover, NL tends to relapse, with a recurrence rate of nearly 50% at 10 years [4]. It is reported that NL causes a massive burden on the healthcare system in the United States (US), with healthcare expenditure of more than 2 billion dollars per year [5]. Therefore, it is necessary to identify the factors that influence NL prevalence in the population. Insulin resistance (IR) is characterized by a decreased responsiveness to glucose disposal of insulin or other metabolic processes mediated by insulin [6]. Gradually increasing evidence demonstrated that IR significantly affects the incidence and progression of multiple disorders, including NL [7,8]. The association between IR and NL may be attributed to urinary acidification, alteration of ionic transportation, and exaggerated inflammatory response at the renal tubular epithelium [9,10]. Although both hyper-insulinemic-euglycemic (HIEG) clamp and homeostasis model assessment for IR (HOMA-IR) are considered the methods for evaluating IR accurately, their procedures are challenging to conduct and time-consuming, limiting widespread use in clinical practice [11]. Recent investigations have proposed several more straightforward indexes for assessing IR, by combining blood lipid and glucose data, such as metabolic syndrome-IR (METS-IR), visceral adiposity (VAI), triglyceride-glucose (TyG), and body mass (BMI) indexes. Several cross-sectional studies found that HOMA-IR and TyG-BMI were significantly related to NL history [7,12,13], and Liu et al. reported similar results in their analysis of TyG-related indices [14]. However, their predictive power for NL appears to be unsatisfactory in some situations due to the underlying factors influencing measurement [7,15]. A novel indicator, estimated glucose disposal rate (eGDR), has been proven by previous evidence to be a dependable substitute for an IR marker. This is related to a lower risk of many disorders with its increase [16,17]. It is also convenient to calculate based on the hypertension status, glycosylated hemoglobin (HGB) level, and waist circumference. Zhang et al. conducted a prospective cohort study and found that eGDR is more reliable in predicting the cardiovascular disease incidence in the Chinese population without diabetes [18]. Compared with other IR markers, eGDR may offer unique advantages for evaluating the association between NL and IR. First, eGDR is simple and feasible to calculate using routinely measured clinical variables. Second, it is less dependent on fasting insulin levels, thus reducing variability associated with insulin assays. Third, its clinical practicality allows for broader implementation in routine metabolic risk assessment. For these reasons, eGDR represents a practical and informative marker to clarify the relationship between IR and NL, and may outperform other indices in population-based and clinical settings.

However, a thorough assessment targeting the relationship between eGDR and NL prevalence has not been performed. Consequently, our study aimed to estimate precisely the relationship between eGDR and NL. We used the propensity score matching (PSM) to control for confounding bias better. Our data were collected from the National Health and Nutrition Examination Survey database (NHANES). NHANES uses a stratified, multistage, clustered probability sampling design that systematically recruits participants from the non‑institutionalized civilian U.S. population across all age, sex, racial, and geographic groups. This rigorous sampling framework ensures that the study cohort closely mirrors the demographic, socioeconomic, and health characteristics of the general United States population. With appropriate survey weighting, analyses using NHANES yield nationally representative results that are generalizable to the broader US population. The hypothesis we proposed was that NL prevalence would decrease as eGDR increases.

## Methods

### Data gathering and filtering

**Figure 1.**
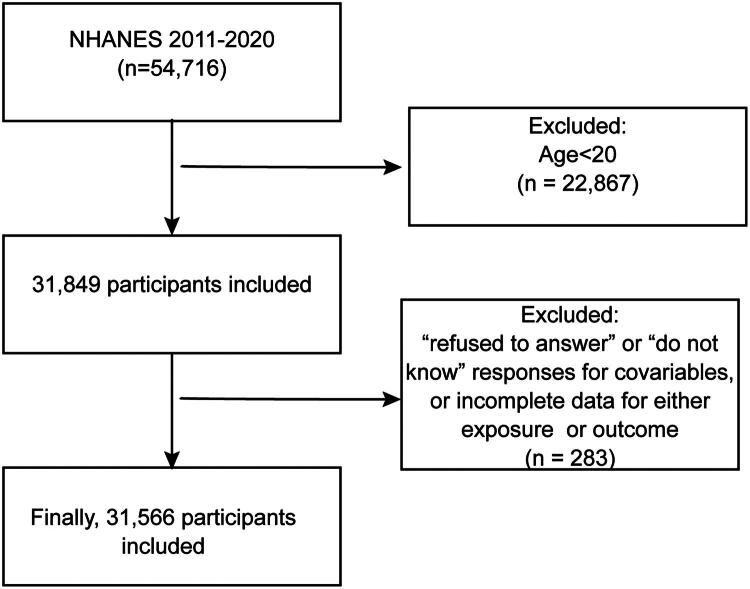
Flowchart on the participant selection.

We gathered data from multiple cycles in the NHANES database from 2011–2020. Complete data were publicly extracted from the official NHANES website (https://www.cdc.gov/nchs/nhanes/index.htm). In total, 54,716 individuals were included in the first instance, then 22,867 whose age did not reach 20 were excluded from the study, and the other 283 were removed from the study, owing to ‘refused to answer’ or ‘do not know’ responses for covariables, or incomplete data for either the exposure (waist circumference, glycosylated hemoglobin A1c, and hypertension) or the outcome (NL). Finally, 31,566 individuals were included in the current study (Figure 1). Missing covariate data were handled by multiple imputation using chained equations (10 imputed datasets, ‘mice’ package in R software, Version 4.4.1), with results combined by Rubin’s rules. The proportion of missing data for each variable is detailed in the (supplementary material Table S1). Briefly, the missing data rates for main covariates ranged from 0% to 57.8%: the lowest missing rate (0%) was observed for age, sex, high blood pressure, race, education, race, marital status, diabetes mellitus, kidney stone, weak kidney and physical activity, while the highest was observed for LDL (57.8%), smoking (57.7%), triglyceride (57.3%) and fasting blood glucose (56.2%). No variable had a missing rate exceeding 60%, indicating a moderate level of missingness overall, which is appropriately addressed by multiple imputation.

### Exposure and outcome determination

The eGDR, usually used to assess insulin resistance, was treated as the exposure variable in this study. To calculate the eGDR of everyone, several parameters, including waist circumference (WC), glycosylated hemoglobin A1c (HbA1c), and hypertension history, were collected from the data module of physical examination, laboratory, and questionnaires, respectively. The formula for eGDR was as follows: eGDR (mg/kg/min) = 21.158 − (0.09 · WC) − (3.407 · hypertension) − (0.551 · HbA1c) [WC (cm), hypertension (yes = 1/no = 0), and HbA1c (%)]. A Tosoh G8 Glycohemoglobin Analyzer was used to measure HbA1c. The iliac crest was considered the location for measuring the WC in a standing position. The question to determine hypertension was, ‘Have you ever been told you have high blood pressure?’ We used the kidney stone history in this study as the outcome variable. Participants were selected based on the response to the question ‘Ever had kidney stones?,’ and we could determine whether they had an NL history. Finally, each was classified into two groups (NL and non-NL groups) based on their selection. No truncation or winsorization was applied to any variables included in the calculation, and individuals with extreme values of waist circumference or HbA1c were not excluded as outliers, to preserve the full nationally representative sample and avoid selection bias in this population-based study.

### Covariates

Furthermore, some indexes obtained from other data modules in the NHANES database were considered covariates in this research. Covariates were selected based on *a priori* biological and epidemiological rationale and previous literature linking IR and NL. Anthropometric measures (age, sex, BMI, waist circumference, etc.) were included as established determinants of metabolic status and stone formation. Metabolic variables (blood pressure, lipid profile, HbA1c, etc.) were adjusted for given their close association with IR and NL. Inflammatory markers such as high-sensitivity C-reactive protein (HS-CRP), were included to account for systemic inflammation. Socioeconomic and lifestyle factors (education, income, lifestyle behaviors) were added to minimize confounding by social and behavioral determinants of NL. The continuous covariates included age, BMI, systolic blood pressure (SBP), diastolic blood pressure (DBP), HGB, fasting plasma glucose (FPG), serum of total cholesterol (TC), low-density lipoproteins (LDL), triglycerides (TG), high-density lipoproteins (HDL), calcium (CA), uric acid (UA), creatinine (CR), and high-sensitivity C-reactive protein (HS-CRP). BMI was calculated as weight in kilograms divided by height in meters squared and then rounded to one decimal place. The third oscillometric reading of blood pressure was recorded as SBP and DBP using a digital upper-arm electronic measurement device. The mobile examination center (MEC) of NHANES produced complete blood counts for blood specimens. The biochemistry profile (including FPG, TC, TG, LDL, HDL, CA, UA, and CR) was measured on the Roche Cobas chemistry analyzer, while HS-CRP was measured on Beckman Coulter UniCel DxC 660 Synchron chemistry analyzers.

The categorical variables were sex, race, education level, marital status, physical activity, household income-to-poverty ratio (PIR), weak kidneys, diabetes mellitus (DM), smoking, and alcohol consumption. Among the above variables, weak kidneys, hypertension, and DM were sorted depending on the answers to the question ‘Ever been told you have weak or failing kidneys except for kidney stones, bladder infections, or incontinence/hypertension/DM?’. For alcoholic drinks, individuals were classified into two groups (< 15 drinks and 15 drinks or more) based on the number of alcoholic drinks consumed during the past 12 months. Physical activity consisted of vigorous, moderate, and other/no intensity activities. Smoking was categorized into no-smoking (do not smoke at all) and smoking (smoke some days or every day) according to the questionnaire ‘Do you now smoke cigarettes?’ Marital status was classified as single (including never married, separated, widowed, and divorced), living with a partner, or married. PIR was converted into three groups: ≥ 3.5, from 1.5–3.499, and no more than 1.5. Moreover, we set a new categorical variable recorded as ‘eGDR (categorical)’ based on each quartile (Q) of eGDR (Q1–Q4).

### Statistical analysis

All analyses accounted for the complex sampling design of NHANES. Survey weights were correctly applied across pooled cycles: cycle-specific MEC weights were used to adjust for sampling probabilities, non-response bias, and the stratified, clustered sampling framework of NHANES. MEC weights, encoded as ‘WTMEC2YR,’ were selected because the primary variables (eGDR, and other laboratory data) were collected during the MEC examination, and these weights ensure the study results are representative of the U.S. civilian non-institutionalized population. According to the methods proposed by Heeringa et al. concerning the combination of weights across cycles [19], the formula for calculating the weight after pooling cycles was as follows: New weight = Weight of the corresponding cycles · Years of the corresponding year/Total years. So, all analyses accounted for the complex NHANES sampling design using WTMEC2YR weights, and consistent with Heeringa et al. weights were appropriately adjusted for pooling across 10 survey cycles to ensure population representativeness. We set a two-tailed *P* value of < 0.05 as statistical significance. Binomial or multinomial variables were presented as counts and proportions. Continuous variables are presented as mean and standard deviation (SD). The weighted Pearson’s chi-square (Rao and Scott adjustment) and design-based Kruskal–Wallis tests were used to compare the binomial data and complex survey samples, respectively. Three models were built in this research using multivariable logistic regression to assess the relevance between eGDR and NL history. Model 1 was adjusted for no covariates. Model 2 was adjusted for sex, race, age, education level, marital status, physical activity, and PIR. Model 3 was adjusted for covariates in model 2, as well as smoking, alcohol consumption, DM, weak kidneys, TC, TG, LDL, HDL, HSCRP, FPG, HGB, CA, CR, and UA. We also investigated the potential relationship between continuous eGDR and the odds ratio (OR) of NL presence in models 1 and 3 with restricted cubic spline (RCS) curve construction. Additionally, stratified by DM, BMI, age, smoking, sex, and race, a subgroup analysis in model 3 was applied to estimate the association and interaction effect between the eGDR (categorical) and NL occurrence. To reduce the impact of age, race, education level, sex, marital status, PIR, physical activity, smoking, DM, LDL, HDL, HGB, CA, UA, and CR, we carried out a 1:1 PSM analysis. We used a logistic regression model to estimate the propensity score, with NL history as the dependent variable and the prespecified covariates as independent variables. These covariates were selected based on basic demographic and clinical potential confounders. We performed 1:1 nearest-neighbor matching without replacement, using a caliper width of 0.2. Covariate balance was assessed using standardized mean differences (SMD), with an absolute SMD < 0.1 considered indicative of adequate balance. Consistent with most observational studies using PSM for baseline confounding adjustment, survey weights were not incorporated into the matching procedure. And we applied the identical statistical analyses to the population after PSM. Finally, to compare the predictive ability of three indicators reflecting IR, receiver operating characteristic (ROC) curves of VAI, BMI, TyG index, and eGDR were generated. R software (Version 4.4.1) (http://www.R-project.org) was utilized for all statistical analyses.

## Results

### Baseline characteristics of participants

In total, 31,566 participants were included in the present study, and the data were obtained from the 2011–2020 NHANES datasets. The group with NL history contained 2,998 individuals, while 28,568 were included in the group without NL history. The eGDR of participants with NL history was lower than the participants without NL history (6.84 ± 2.75 mg/kg/min against 8.02 ± 2.68 mg/kg/min, *p* < 0.001). Compared to the group without NL history, the participants with NL history had higher BMI, waist, FPG, LDL, HGB, HbA1c, UA, CR, HS-CRP, SBP, DBP, and lower HDL and CA levels. Additionally, participants with an NL history had a higher proportion of males, vigorous physical activity, weak kidneys, hypertension, and DM, and were more likely to be older. There were no statistically significant differences between the two groups regarding education, PIR, TC, TR, and alcohol consumption. weighted means were used for all descriptive statistics, in accordance with standard NHANES practice. Detailed information comparing baseline characteristics between the two groups is listed in Table 1. Supplementary Material (Table S1) reports missing data before multiple imputations.

**Table 1.** Baseline characteristics of participants before PSM and after PSM.

	history of nephrolithiasis (before PSM)			history of nephrolithiasis (after PSM)		
Characteristic	No *n* = 28,568^a^	Yes *n* = 2,998^a^	*P* ^b^	No *n* = 2,998^a^	Yes *n* = 2,998^a^	*P* ^b^
**Sex**			0.003			0.7609
Male	13,676 (48%)	1,603 (53%)		1,591 (53%)	1,603 (53%)	
Female	14,892 (52%)	1,395 (47%)		1,407 (47%)	1,395 (47%)	
**Age**	47± (17)	53± (16)	<0.001	53± (17)	53± (16)	0.8
**BMI (kg/m^2^)**	29± (7)	31± (7)	<0.001	30± (7)	31± (7)	<0.001
**Waist(cm)**	100± (17)	105± (17)	<0.001	103± (17)	105± (17)	<0.001
**Systolic blood pressure (mmHg)**	122± (18)	124± (18)	<0.001	123± (18)	124± (18)	0.034
**Diastolic blood pressure (mmHg)**	71± (12)	72± (13)	0.004	71± (12)	72± (13)	0.006
**Hemoglobin (g/dL)**	14.14± (1.47)	14.22±(1.47)	0.007	14.19± (1.47)	14.22± (1.47)	0.3
**HbA1c (%)**	5.64± (0.94)	5.86± (1.08)	<0.001	5.79± (1.03)	5.86± (1.08)	0.013
**Fasting blood glucose (mg/dL)**	108± (31)	116± (37)	<0.001	113± (36)	116± (37)	<0.001
**HDL (mg/dL)**	54± (17)	51± (15)	<0.001	52± (16)	51± (15)	0.2
**HS-CRP (mg/dL)**	3.9± (7.9)	4.7± (8.8)	<0.001	4.0± (6.8)	4.7± (8.8)	<0.001
**Creatinine (mg/dL)**	0.88± (0.35)	0.94± (0.59)	<0.001	0.94± (0.57)	0.94± (0.59)	0.6
**Calcium (mg/dL)**	9.36± (0.36)	9.33± (0.38)	0.004	9.34± (0.36)	9.33± (0.38)	0.3
**Uric acid (mg/dL)**	5.36± (1.42)	5.53± (1.46)	<0.001	5.46± (1.42)	5.53± (1.46)	0.2
**Total cholesterol (mg/dL)**	191± (41)	190± (44)	0.6	191± (42)	190± (44)	0.8
**Triglyceride (mg/dL)**	221± (205)	224± (209)	0.4	230± (210)	224± (209)	0.4
**LDL (mg/dL)**	93± (45)	95± (45)	0.032	94± (45)	95± (45)	0.3
**15 drinks or more past 12 months**			0.052			0.067
No	28,410 (99%)	2,991 (100%)		2,980 (99%)	2,991 (100%)	
Yes	158 (0.5%)	7 (0.2%)		18 (0.6%)	7 (0.2%)	
**High blood pressure**			<0.001			<0.001
No	18,328 (69%)	1,454 (52%)		1,670 (56%)	1,454 (48%)	
Yes	10,240 (31%)	1,544 (48%)		1,328 (44%)	1,544 (52%)	
**Race**			<0.001			0.8
Mexican American	3,701 (8.8%)	357 (6.5%)		376 (13%)	357 (12%)	
Other Hispanic	2,903 (6.7%)	353 (6.1%)		355 (12%)	353 (12%)	
Non-Hispanic White	9,970 (63%)	1,470 (74%)		1,478 (49%)	1,470 (49%)	
Non-Hispanic Black	7,064 (12%)	458 (6.5%)		455 (15%)	458 (15%)	
Other Race	4,930 (9.4%)	360 (7.2%)		334 (11%)	360 (12%)	
**Marital status**			<0.001			0.8
Living with a partner or married	16,511 (62%)	1,859 (68%)		1,843 (61%)	1,859 (62%)	
Single	12,057 (38%)	1,139 (32%)		1,155 (39%)	1,139 (38%)	
**Education**			0.6			0.5
Less than high school	6,060 (14%)	678 (14%)		719 (24%)	678 (23%)	
High school	6,532 (23%)	660 (23%)		683 (23%)	660 (22%)	
Above high school	15,976 (63%)	1,660 (63%)		1,596 (53%)	1,660 (55%)	
**Diabetes mellitus**			<0.001			0.6
No	24,780 (90%)	2,287 (81%)		2,310 (77%)	2,287 (76%)	
Yes	3,788 (9.7%)	711 (19%)		688 (23%)	711 (24%)	
**Weak kidney**			<0.001			<0.001
No	27,629 (97%)	2,721 (93%)		2,817 (94%)	2,721 (91%)	
Yes	939 (2.6%)	277 (6.8%)		181 (6.0%)	277 (9.2%)	
**Physical activity**			0.014			0.9
Vigorous	16,564 (53%)	17,18 (53%)		1,750 (58%)	1,718 (57%)	
Moderate	6,045 (24%)	592 (21%)		573 (19%)	592 (20%)	
Other or no	5,959 (23%)	688 (26%)		675 (23%)	688 (23%)	
**Smoking**			<0.001			0.4
Never	15,295 (57%)	1,829 (64%)		1,867 (62%)	1,829 (61%)	
Some days or every day	13,273 (43%)	1,169 (36%)		1,131 (38%)	1,169 (39%)	
**PIR**			0.3			0.8
<1.5	10,641 (26%)	1,108 (25%)		1,132 (38%)	1,108 (37%)	
1.5-3.499	9,359 (31%)	983 (31%)		987 (33%)	983 (33%)	
≥3.5	8,568 (42%)	907 (44%)		879 (29%)	907 (30%)	
**Categorical eGDR**			<0.001			<0.001
Q1	7,597 (23%)	12,23 (39%)		737 (25%)	873 (29%)	
Q2	7,340 (25%)	878 (28%)		801 (27%)	879 (29%)	
Q3	6,801 (26%)	543 (19%)		703 (23%)	688 (23%)	
Q4	6,830 (26%)	354 (14%)		757 (25%)	558 (19%)	
**Continuous eGDR**	8.02± (2.68)	6.84± (2.75)	<0.001	7.46± (2.76)	6.84± (2.75)	<0.001

^a^n (unweighted) (%) for categorical variables; Mean± (SD) for continuous variables.

^b^Pearson’s X^2: Rao & Scott adjustment for binomial data; Design-based Kruskal Wallis test for complex survey samples.

BMI, Body mass index; eGDR, Estimated glucose disposal rate; HbA1c, Glycosylated hemoglobin A1c; HDL, High-density lipoprotein; HS-CRP, High-sensitivity c-reactive protein; LDL, Low-density lipoprotein; PIRHousehold income–to–poverty ratio; PSM, Propensity score matching; SD standard deviations.

### Association between eGDR and NL

**Figure 2.**
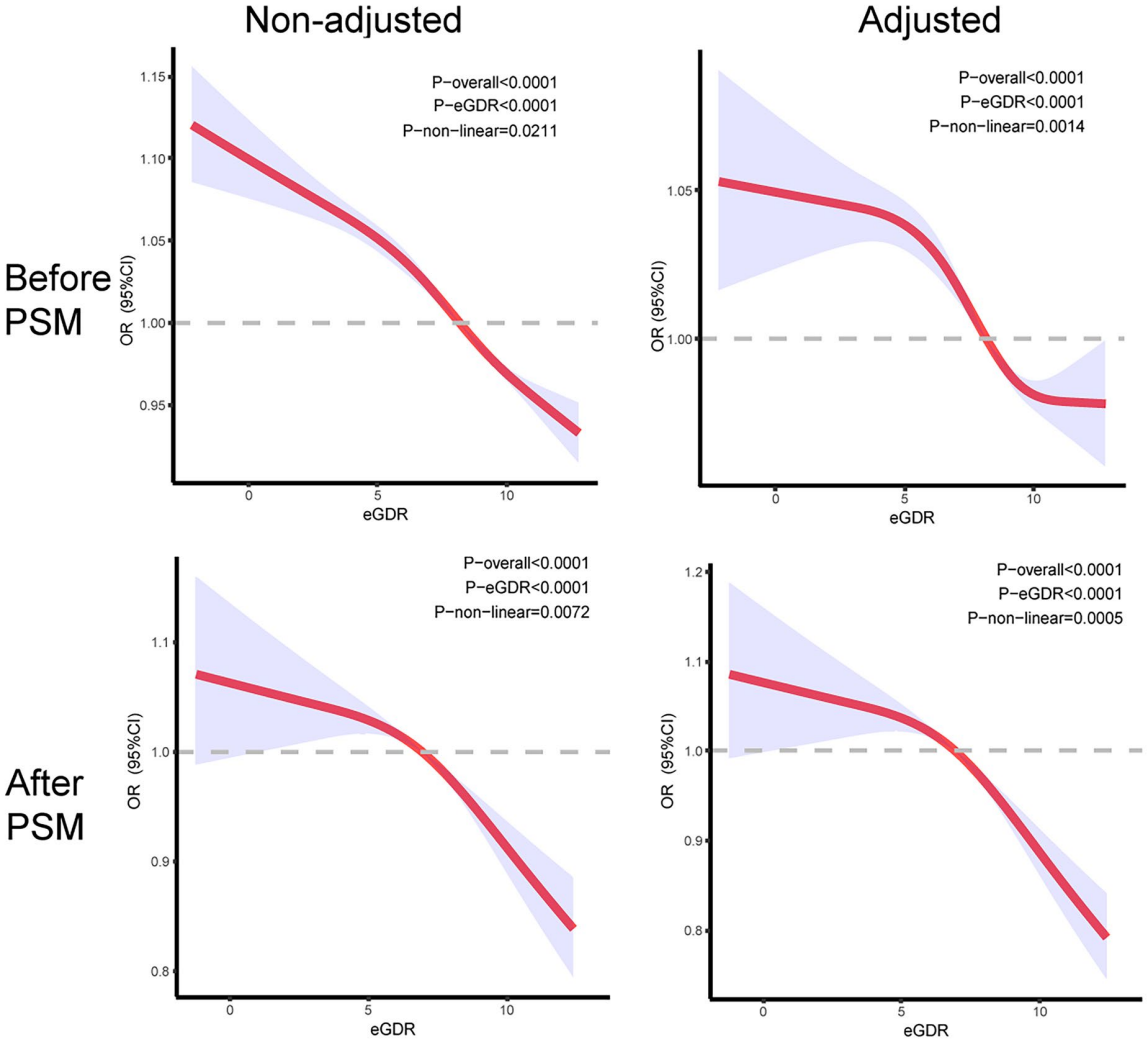
RCS curves for CVD according to the eGDR before and after PSM. The adjusted models adjusted covariates in model 3, including sex, race, age, education level, marital status, physical activity, PIR, smoking, alcoholic consumption, diabetes, weak kidneys, TC, TG, LDL, HDL, HSCRP, FPG, HGB, CA, CR, and UA.

Three models were constructed to assess the association between eGDR and NL using weighted multivariable logistic regression. A significant negative relevance was observed in each model (Table 2). After adjusting for the covariates in model 3, we discovered each unit increment in eGDR related to a 10% decrease in NL prevalence [OR = 0.90, 95% confidence interval (CI): 0.88–0.93, *p* < 0.001]. Furthermore, for the Q1 of categorical eGDR sorted by its Q, the adjusted ORs (95% CI) of Q2–Q4 in model 3 were as follows: 0.83 (0.68 and 1.00), 0.59 (0.48 and 0.71), and 0.50 (0.39 and 0.64). Figure 2 illustrates RCS curves between eGDR and the OR of NL. Although the p-values for nonlinearity were statistically significant, we concluded linearity based on visual inspection of the fitted curves, which showed an approximately linear trend without obvious departure from linearity. The results suggested that there might be an inverse linear correlation between the NL history and continuous eGDR before or after adjusting for covariates in model 3 (all *P*-overall < 0.0001, all *P*-eGDR < 0.0001, before adjusting *P* for nonlinear = 0.0211, after adjusting *P* for nonlinear = 0.0014). We also included the components of the eGDR formula (HbA1c, waist circumference, and hypertension) in the analysis. The results are illustrated in the Supplementary Materials (Tables S2 and S3), indicating that eGDR can provide an independent predictive value of NL.

**Table 2.** Weighted multivariable logistic regression for the association between the eGDR and NL risk before PSM.

	OR (95%CI), P-value		
	model 1	model2	model3
**Continuous eGDR**	0.86 (0.84, 0.88) <0.001	0.88 (0.86, 0.90) <0.001	0.90 (0.88, 0.93) <0.001
**Categorical eGDR**			
Q1	Reference	Reference	Reference
Q2	0.69 (0.58, 0.81) <0.001	0.74 (0.62, 0.88) <0.001	0.83 (0.68, 1.00) 0.045
Q3	0.45 (0.38, 0.53) <0.001	0.51 (0.42, 0.60) <0.001	0.59 (0.48, 0.71) <0.001
Q4	0.31 (0.26, 0.38) <0.001	0.40 (0.32, 0.50) <0.001	0.50 (0.39, 0.64) <0.001

Model 1 was adjusted for no covariates. Model 2 was adjusted for age, sex, race, physical activity, education level, marital status, and PIR. Model 3 was adjusted for covariates in model 2 + smoking, drink, diabetes mellitus, total cholesterol, triglyceride, LDL, HDL, HS-CRP, fasting blood glucose, hemoglobin, weak kidney, calcium, and creatinine.

**Table 3.** Subgroup analyses between categorical eGDR and NL risk in model 3 before PSM.

Subgroups	OR (95%CI), *P*-value				*P* for interaction
	Q1	Q2	Q3	Q4	
**Age**					0.12
<40	reference	0.82 (0.60, 1.11) 0.2	0.68 (0.49, 0.94) 0.022	0.54 (0.39, 0.76) <0.001	
40-59	reference	0.76 (0.56, 1.02) 0.07	0.46 (0.34, 0.62) <0.001	0.37 (0.25, 0.55) <0.001	
≥60	reference	0.92 (0.73, 1.15) 0.4	0.68 (0.52, 0.90) 0.007	0.75 (0.41, 1.38) 0.3	
**Sex**					0.06
Female	reference	0.86 (0.70, 1.05) 0.13	0.65 (0.52, 0.82) <0.001	0.49 (0.35, 0.69) <0.001	
Male	reference	0.83 (0.62, 1.12) 0.2	0.55 (0.43, 0.72) <0.001	0.54 (0.40, 0.73) <0.001	
**Race**					0.052
Mexican American	reference	1.34 (0.94, 1.91) 0.11	0.77 (0.49, 1.21) 0.2	0.61 (0.34, 1.08) 0.089	
Other Hispanic	reference	0.98 (0.63, 1.53) >0.9	0.6 (0.38, 0.96) 0.032	0.53 (0.26, 1.07) 0.077	
Non-Hispanic White	reference	0.82 (0.65, 1.03) 0.084	0.61 (0.48, 0.78) <0.001	0.51 (0.37, 0.71) <0.001	
Non-Hispanic Black	reference	0.74 (0.59, 0.94) 0.014	0.55 (0.39, 0.79) 0.002	0.65 (0.42, 1.00) 0.051	
Other Race	reference	0.74 (0.38, 1.42) 0.4	0.42 (0.25, 0.71) 0.002	0.37 (0.20, 0.70) 0.003	
**Diabetes mellitus**					0.04
Yes	reference	1.06 (0.76, 1.47) 0.7	0.31 (0.17, 0.54) <0.001	0.83 (0.16, 4.23) 0.8	
No	reference	0.79 (0.65, 0.94) 0.011	0.60 (0.48, 0.74) <0.001	0.51 (0.39, 0.66) <0.001	
**BMI**					0.36
<25	reference	1.00 (0.59, 1.17) >0.9	0.73 (0.40, 1.32) 0.3	0.57 (0.30, 1.10) 0.09	
25-29	reference	1.00 (0.76,1.31) >0.9	0.66 (0.48, 0.89) 0.008	0.73 (0.44, 1.21) 0.2	
≥30	reference	0.80 (0.63, 1.02) 0.072	0.62 (0.46, 0.83) 0.002	0.22 (0.06, 0.80) 0.023	
**Smoking**					0.84
Some days or every day	reference	0.84 (0.65, 1.08) 0.2	0.61 (0.45, 0.83) 0.002	0.56 (0.39, 0.81) 0.003	
Never	reference	0.83 (0.66, 1.03) 0.087	0.58 (0.44, 0.76) <0.001	0.46 (0.33, 0.64) <0.001	

BMI, Body mass index; eGDR, Estimated glucose disposal rate; NL, Nephrolithiasis; PSM, Propensity score matching.

### Subgroup analysis

We performed a subgroup analysis in model 3 to assess whether the association between eGDR and NL was stable throughout diverse stratifications. As illustrated in Table 3, only the diabetes subgroups were found to have a significant interaction on the association between categorical eGDR and NL (*P* for interaction = 0.04). A significant negative relationship between categorical eGDR and NL, which is similar to the primary outcome, was observed among most subgroups. The never-smoking subgroup had a lower NL prevalence than the smoking subgroup (smoked some days or every day) with an eGDR increase (Q4 in never-smoking, OR = 0.46; Q4 in smoking, OR = 0.56).

### Analysis after PSM

**Figure 3.**
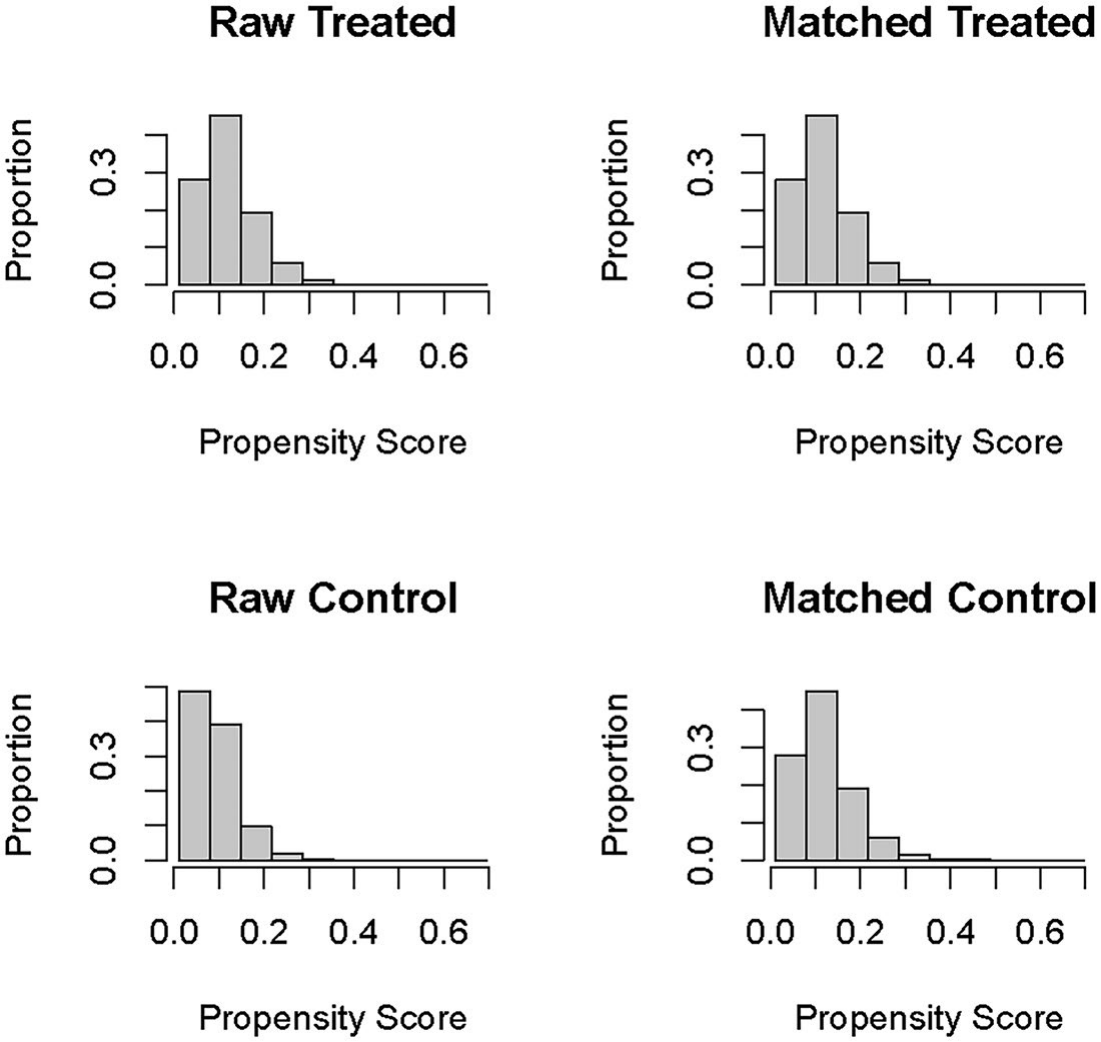
Propensity score distribution of groups before PSM and after PSM.

After PSM, all covariates achieved satisfactory balance with an absolute SMD of less than 0.1. Propensity score histograms (Figure 3) also demonstrated good overlap between groups, supporting the validity of the matching procedure. Following PSM, a total of 5,996 participants were included in the analysis, comprising 2,998 participants with a history of NL and 2,998 participants without a history of NL. Table 1 demonstrates no significant difference between these two groups in terms of sex, race, age, education level, marital status, physical activity, PIR, smoking, DM, LDL, HDL, HGB, CA, UA, and CR after PSM. We used the weighted multivariable logistic regression to analyze participants after PSM and construct the models adjusted for the covariates as before. As listed in Table 4, NL prevalence was negatively associated with both continuous and categorical eGDR (OR of continuous eGDR = 0.90, 95% CI: 0.86–0.93, *p* < 0.001; when compared to Q1 of categorical eGDR, OR of Q4 = 0.41, 95% CI: 0.31–0.55, *p* < 0.001). Figure 2 also illustrates an inverse linear correlation between continuous eGDR and the OR of NL formation, with or without adjusting for covariates in model 3 (all *P*-overall < 0.0001, all *P*-eGDR < 0.0001, non-adjusting *P*-non-linear = 0.0072, adjusting *P*-non-linear = 0.0005). Table 5 displays the results of the subgroup analysis of the participants after PSM. We observed an interaction effect on the association between eGDR and NL formation in BMI subgroups (*P* for interaction = 0.04), and a significant and negative relationship between categorical eGDR and NL remained in most subgroups.

**Table 4.** Weighted multivariable logistic regression for the association between the eGDR and NL risk after PSM.

	OR (95%CI), P-value		
	model 1	model 2	model 3
Continuous eGDR	0.92 (0.90, 0.95) <0.001	0.91 (0.88, 0.93) <0.001	0.90 (0.86, 0.93) <0.001
Categorical eGDR			
Q1	Reference	Reference	Reference
Q2	0.81 (0.68, 0.96) 0.015	0.79 (0.67, 0.93) 0.006	0.76 (0.63, 0.92) 0.006
Q3	0.65 (0.53, 0.81) <0.001	0.61 (0.49, 0.76) <0.001	0.59 (0.45, 0.78) <0.001
Q4	0.51 (0.41, 0.62) <0.001	0.45 (0.36, 0.56) <0.001	0.41 (0.31, 0.55) <0.001

Model 1 was adjusted for no covariates. Model 2 was adjusted for age, sex, race, physical activity, education level, marital status, and PIR. Model 3 was adjusted for covariates in model 2 + smoking, drink, diabetes mellitus, total cholesterol, triglyceride, LDL, HDL, HS-CRP, fasting blood glucose, hemoglobin, weak kidney, calcium, and creatinine.

**Table 5.** Subgroup analyses between categorical eGDR and NL risk in model 3 after PSM.

Subgroups	OR (95%CI), *P*-value				*P* for interaction
	Q1	Q2	Q3	Q4	
**Age**					0.7
<40	reference	0.56 (0.29, 1.05) 0.071	0.57 (0.31, 1.06) 0.075	0.42 (0.24, 0.74) 0.003	
40-59	reference	0.66 (0.44, 1.01) 0.056	0.48 (0.29, 0.79) 0.005	0.33 (0.19, 0.58) <0.001	
≥60	reference	0.87 (0.68, 1.12) 0.3	0.62 (0.44, 0.89) 0.009	0.53 (0.33, 0.86) <0.011	
**Sex**					0.054
Female	reference	0.81 (0.61, 1.07) 0.14	0.71 (0.49, 1.03) 0.069	0.40 (0.27,0.60) <0.001	
Male	reference	0.74 (0.53, 1.02) 0.063	0.52 (0.36, 0.73) <0.001	0.44 (0.29, 0.67) <0.001	
**Race**					0.14
Mexican American	reference	1.26 (0.77, 2.06) 0.3	1.33 (0.75, 2.36) 0.3	0.90 (0.45, 1.08) 0.8	
Other Hispanic	reference	0.69 (0.40, 1.18) 0.2	0.58 (0.33, 1.02) 0.06	0.39 (0.16, 0.96) 0.04	
Non-Hispanic White	reference	0.71 (0.55, 0.91) 0.007	0.55 (0.40, 0.77) <0.001	0.38 (0.26, 0.54) <0.001	
Non-Hispanic Black	reference	0.98 (0.61, 1.55) >0.9	0.64 (0.38, 1.09) 0.10	0.85 (0.46, 1.57) 0.6	
Other Race	reference	0.58 (0.28, 1.17) 0.12	0.64 (0.26, 1.59) 0.3	0.29 (0.12, 0.69) 0.006	
**Diabetes mellitus**					0.32
Yes	reference	1.11 (0.72, 1.71) 0.6	0.62 (0.36, 1.05) 0.075	0.24 (0.05, 1.20) 0.081	
No	reference	0.69 (0.53, 0.89) 0.005	0.57 (0.42, 0.78) <0.001	0.41 (0.29, 0.56) <0.001	
**BMI**					0.04
<25	reference	1.01 (0.16, 6.38) >0.9	0.73 (0.12, 4.36) 0.7	0.59 (0.09, 3.83) 0.6	
25-29	reference	0.73 (0.47, 1.14) 0.2	0.53 (0.34, 0.83) 0.007	0.29 (0.16, 0.52) <0.001	
≥30	reference	0.73 (0.56, 0.95) 0.019	0.60 (0.43, 0.84) 0.004	1.39 (0.74, 2.59) 0.3	
**Smoking**					0.3
Some days or every day	reference	0.72 (0.54, 0.96) 0.024	0.71 (0.52, 0.99) 0.045	0.47 (0.32, 0.71) <0.001	
Never	reference	0.79 (0.61, 1.02) 0.066	0.54 (0.38, 0.76) <0.001	0.36 (0.25, 0.53) <0.001	

BMI, Body mass index; DM, Diabetes mellitus; eGDR, Estimated glucose disposal rate; NL, Nephrolithiasis; PSM, Propensity score matching.

### Predictive ability of eGDR

**Figure 4.**
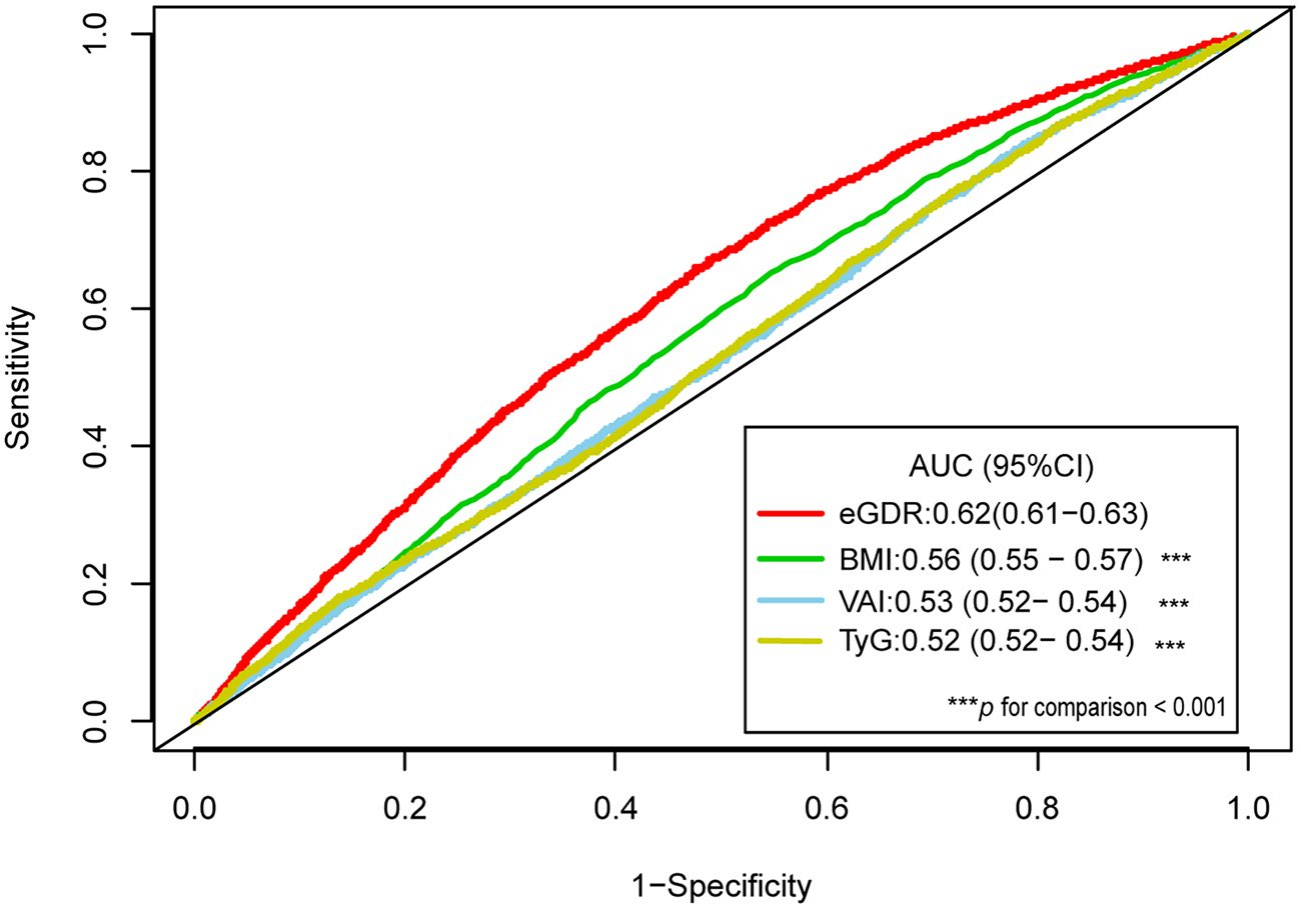
ROC curve for predicting the NL incidence.

By generating ROC curves to determine the predictive capability of the TyG, VAI, BMI, and eGDR, Figure 4 suggests that the area under the curve (AUC) of BMI, TyG, and VAI was smaller than that of eGDR. The AUC values were 0.62 for eGDR, 0.56 for BMI, 0.52 for TyG, and 0.53 for VAI, with all pairwise comparison *P*-values < 0.001.

## Discussion

We performed this study to determine a relationship between eGDR and NL history, covering 31,566 individuals representing a national population older than 20 years in the US. After adjusting for the covariates in model 3, a higher eGDR, which reflects a lower IR, was significantly related to a lower NL prevalence. RCS results suggest that there exists an inverse linear correlation between NL history and continuous eGDR, and the trends of RCS curves were consistent with multivariable logistic regression analysis. Subgroup analysis also revealed that the negative relationship was stable among various subgroups. Moreover, to control confounding bias further, a similar analysis was performed after PSM, and the main results of multivariate logistic regression, RCS, and subgroup analysis remained the same. ROC curves illustrated that eGDR was better at discriminating people with NL than VAI or BMI. These results provide further support for the hypothesis that eGDR might be an auspicious parameter for predicting NL, and managing the level of eGDR may help reduce the NL occurrence. In summary, the present study has several notable strengths. First, this study was based on a large and nationally representative sample from the NHANES, which provides strong reliability and enhances the generalizability of the findings. Second, we adopted robust and rigorous statistical methods, including PSM, RCS analysis, and survey weight adjustment, to minimize confounding bias and improve the validity of the results. Third, we comprehensively compared the performance of eGDR with multiple widely used IR surrogates, which helps to clarify its incremental value and practical implications.

Extensive research has demonstrated that IR plays a role in developing various diseases, such as atherosclerosis, DM, chronic kidney disease, obesity, and osteoporosis [20–24]. Prior studies have also noted the IR importance in NL formation. Ando et al. conducted a cross-sectional analysis in the NHANES database and used the HOMA-IR to represent the insulin resistance level. They discovered that the significant OR of developing NL could be 2.11, with elevated HOMA-IR [25]. And our findings extend those reported by them. Compared to HOMA-IR, which requires fasting blood samples and insulin measurements, eGDR is a practical, non-fasting index based on routinely available clinical measures including WC, HbA1c, and hypertension. This renders eGDR more convenient and feasible in large-scale epidemiological studies and clinical settings where insulin levels or fasting conditions are not always available. Similarly, Ando et al. included 1036 healthy Japanese subjects in their study. After collecting overnight fasting blood of the participants to measure insulin and calculate HOMA-IR, we found that HOMA-IR and insulin were significantly associated with NL history in females [26]. A retrospective cohort study by Kim et al. illustrated that with the glycemic values and HOMA-IR, the risk for NL, especially in the male population, would increase [10]. Recently, researchers have investigated various IR makers to assess and better manage IR. Data from several previous studies illustrated that HOMA-IR is a reliable indicator for predicting NL [27,28]. Moreover, METS-IR and TyG indexes, based on fasting blood lipid and glucose data, are surrogate markers of IR and positively related to the risk of developing NL [13,29]. Additionally, obesity-related indicators, including BMI, weight-adjusted waist index, and VAI, could reflect IR indirectly and are significantly associated with NL [30]. However, there are some limitations in the clinical practice of the indices outlined above. Fasting blood glucose is necessary for calculating the TyG, METS-IR, and HOMA-IR, and many factors, such as diet, medicine, and various stressors, influence it.

Furthermore, their formulas are relatively complicated, and the HOMA-IR calculation even requires fasting blood insulin, which is not a routine examination for the general population[31]. For the obesity-related indicators, their formulas contain only demographic data and blood lipids. Consequently, they cannot comprehensively evaluate the IR of the body and are unlikely to have satisfactory sensitivity and specificity in predicting the NL occurrence [32].

Recently, the eGDR has turned out to be a novel and reliable marker of IR, associated with vascular events and all-cause mortality [33,34]. Zenglei et al. proposed that eGDR, formed by laboratory and clinical indices, is much more reliable and comprehensive for IR evaluation [18]. This superiority may be explained by its inclusion of multiple complementary markers of insulin resistance: WC captures central adiposity, HbA1c reflects chronic glycemic exposure, and hypertension represents a key component of the metabolic milieu linked to both IR and NL pathogenesis. In contrast, BMI reflects only overall adiposity, and TyG focuses primarily on triglyceride and glucose levels. And our ROC results revealed that eGDR has the most significant predictive power among the several common indicators. These findings are also consistent with previous studies supporting the value of composite IR indices that incorporate multiple metabolic domains. Although eGDR demonstrated a significantly higher AUC (0.62) than BMI, TyG, and VAI in our analysis, suggesting superior discriminatory performance, the overall AUC values indicate weak overall discrimination. Thus, the absolute predictive ability of eGDR for kidney stones remains limited, and its clinical utility as a standalone predictive marker may be modest. The clinical applicability and actual diagnostic value of eGDR remain to be verified in larger prospective cohorts and real-world clinical settings.

In subgroup analysis, a more significant relationship between eGDR and NL was observed in the non-DM subgroup than in the DM subgroup. This phenomenon is because few patients with diabetes have a high HbA1c level, and their eGDR was relatively low. Accordingly, the sample sizes in the groups with high eGDR levels (Q4 and Q3) would be small, causing a strong bias. Similar results can be found in the study conducted by Ren et al. suggesting that individuals without diabetes are more sensitive to eGDR [35].

Although more researchers have proposed that IR affects stone formation, the strict biological processes are not fully understood. One widely accepted mechanism is changing the ammonium and pH in renal tubules. A controlled intervention study conducted by Nicola et al. suggested that ammonium and urine pH decreased with increasing IR measured by glucose disposal rate. This manifestation may be due to the reduction in ammonium production and secretion in renal proximal tubules [36]. Eventually, activating the acid-base balance adjusted mechanism leads to UA precipitation and NL [30]. Similar results have been found in studies on rat models of metabolic syndrome [37].

Additionally, patients with IR are more likely to display higher endogenous acid production and lower citrate excretion, contributing to stone formation [38,39]. Another possible mechanism is the immune system disorder caused by IR, which induces the inflammatory response and oxidative stress elevation in the system, including the kidneys. Consequently, urinary excretion of oxalate and CA, along with CA oxalate crystal precipitation, increases [9,40]. As is known to us, WC reflects central adiposity, which is closely linked to chronic low-grade inflammation and oxidative stress. And HbA1c indicates long-term glycemic exposure, which also promotes oxidative damage and inflammatory cascades in the renal microenvironment. So eGDR incorporates both WC and HbA1c, this index indirectly captures the inflammatory and oxidative stress pathways that connect IR to NL. Notably, Vivienne et al. reported no relationship between glucose disposal rate and CA level in 24-h urine or CA fractional excretion in a HIEG clamp study that targeted the population with idiopathic hypercalciuria. IR does not influence the CA stone development through the renal excretion of CA [41]. Further studies are needed to explore the mechanisms underlying the relationship between IR and NL.

There are a few limitations that should be paid attention to. First, the present analysis was based on NHANES data, targeting individuals aged 20 and above in the US. Moreover, the generalizability of our conclusions may be limited in other regions and demographics worldwide. Second, NL status was based on self-reported history of kidney stones. This may introduce recall bias, as some participants may inaccurately report past stone events. In addition, asymptomatic or clinically undetected stones among individuals without a self-reported history could not be identified, which may lead to misclassification of non-case participants. These limitations should be considered when interpreting the observed associations. Third, despite adjusting for many covariates in models, confounding remained in our analysis. These underlying factors influencing the results include drinking water quality, long-term medicine administration, and operation history [42,43]. Fourth, we lacked data on kidney stone composition (e.g., calcium oxalate, uric acid), which may have distinct metabolic associations. Finally, this study employed a cross-sectional design using NHANES data, which precludes determination of temporal sequence and causal inference between eGDR and NL. Because exposure (eGDR) and outcome (NL) were measured at the same time, temporal sequence cannot be confirmed, and it is also difficult to differentiate incident from prevalent NL. We cannot determine whether altered IR precedes stone formation or develops as a consequence of NL-related factors, limiting conclusions about predictive performance for incident NL risk.

## Conclusion

Our results exhibited that a lower eGDR was associated with a higher NL prevalence. eGDR improved the discrimination of NL history compared with TyG, BMI, and VAI, which would help validate the association. However, its predictive value remains modest, indicating that it is not yet sufficient for standalone clinical application. Further validation in large-scale prospective cohorts is therefore warranted to confirm its reliability and clinical utility.

## Supplementary Material

Supplemental Material

Supplemental Material

Supplemental Material

## Data Availability

This study analyzed publicly available datasets. All the raw data employed for this study are obtained from the NHANES repository (https://wwwn.cdc.gov/nchs/nhanes/analyticguidelines.aspx).

## Abbreviations

BMI: Body mass index
CA: Calcium
CI: Confidence interval
CR: Creatinine
DBP: Diastolic blood pressure
DM: Diabetes mellitus
eGDR: Estimated glucose disposal rate
FBG: Fasting blood glucose
HBP: High blood pressure
HbA1c: Glycosylated hemoglobin A1c
HDL: High-density lipoprotein
HGB: Hemoglobin
HIEG: Hyperinsulinemic- euglycemic
HOMA-IR: Homeostasis model assessment for insulin resistance
HS-CRP: High-sensitivity c-reactive protein
IR: Insulin resistance
LDL: Low-density lipoprotein
MEC: Mobile examination center
METS-IR: Metabolic Syndrome-Insulin Resistance
NHANES: National Health and Nutrition Survey
NL: Nephrolithiasis
OR: Odds ratio
PIR: Household income–to–poverty ratio
PSM: Propensity score matching
Q: Quartiles
RCS: Restricted cubic spline
SBP: Systolic blood pressure
SD: Standard deviations
SMD: Standardized mean differences
TC: Total cholesterol
TG: Triglyceride
TyG: Triglyceride-glucose
UA: Uric acid
US: United States
WC: Waist circumference
WWI: Weight-adjusted waist index

## References

[1] Tan S, Yuan D, Su H, et al. Prevalence of urolithiasis in China: a systematic review and meta-analysis. BJU Int. 2024;133(1):34–43. doi: 10.1111/bju.16179. 37696625

[2] Sui W, Hancock J, Asplin JR, et al. Nephrolithiasis and elevated urinary ammonium: a matched comparative study. Urology. 2020;144:77–82. doi: 10.1016/j.urology.2020.05.063. 32544550

[3] Wigner P, GręBowski R, Bijak M, et al. The molecular aspect of nephrolithiasis development. Cells. 2021;10(8):1926. doi: 10.3390/cells10081926. 34440695 PMC8393760

[4] Siener R. Nutrition and kidney stone disease. Nutrients. 2021;13(6):1917. doi: 10.3390/nu13061917. 34204863 PMC8229448

[5] Thongprayoon C, Krambeck AE, Rule AD. Determining the true burden of kidney stone disease. Nat Rev Nephrol. 2020;16(12):736–746. doi: 10.1038/s41581-020-0320-7. 32753740

[6] Hill MA, Yang Y, Zhang L, et al. Insulin resistance, cardiovascular stiffening and cardiovascular disease. Metabolism. 2021;119:154766. doi: 10.1016/j.metabol.2021.154766. 33766485

[7] Shen Y, Zhu Z, Bi X, et al. Association between insulin resistance indices and kidney stones: results from the 2015-2018 National Health and Nutrition Examination Survey. Front Nutr. 2024;11:1444049. doi: 10.3389/fnut.2024.1444049. 39416649 PMC11480067

[8] Wong Y, Cook P, Roderick P, et al. Metabolic syndrome and kidney stone disease: a systematic review of literature. J Endourol. 2016;30(3):246–253. doi: 10.1089/end.2015.0567. 26576717

[9] He Q, Tang Y, Li Y, et al. A pilot dynamic analysis of formative factors of nephrolithiasis related to metabolic syndrome: evidence in a rat model. Ren Fail. 2022;44(1):1134–1143. doi: 10.1080/0886022X.2022.2097922. 35837686 PMC9291672

[10] Kim S, Chang Y, Jung H-S, et al. Glycemic status, insulin resistance, and the risk of nephrolithiasis: a cohort study. Am J Kidney Dis. 2020;76(5):658–668.e1. doi: 10.1053/j.ajkd.2020.03.013. 32534797

[11] Cersosimo E, Solis-Herrera C, Trautmann ME, et al. Assessment of pancreatic β-cell function: review of methods and clinical applications. Curr Diabetes Rev. 2014;10(1):2–42. doi: 10.2174/1573399810666140214093600. 24524730 PMC3982570

[12] Yang YX, Xiang JC, Ye GC, et al. Association of insulin resistance indices with kidney stones and their recurrence in a non-diabetic population: an analysis based on NHANES data from 2007-2018. Ren Fail. 2025;47(1):2490203. doi: 10.1080/0886022X.2025.2490203. 40275575 PMC12035944

[13] Shen X, Chen Y, Chen Y, et al. Is the METS-IR index a potential new biomarker for kidney stone development? Front Endocrinol (Lausanne). 2022;13:914812. doi: 10.3389/fendo.2022.914812. 35909543 PMC9329808

[14] Liu M, Yang P, Gou Y. Association between triglyceride glucose index-related indices and kidney stones in adults based on NHANES 2007-2020. Front Endocrinol (Lausanne). 2024;15:1516982. doi: 10.3389/fendo.2024.1516982. 39839481 PMC11746126

[15] Wang D, Zhang D, Zhang L, et al. Association between triglyceride-glucose index and risk of kidney stone: a Chinese population-based case-control study. BMJ Open. 2024;14(11):e086641. doi: 10.1136/bmjopen-2024-086641. PMC11590796 39578031

[16] Nyström T, Holzmann MJ, Eliasson B, et al. Estimated glucose disposal rate predicts mortality in adults with type 1 diabetes. Diabetes Obes Metab. 2018;20(3):556–563. doi: 10.1111/dom.13110. 28884949

[17] Penno G, Solini A, Orsi E, et al. Insulin resistance, diabetic kidney disease, and all-cause mortality in individuals with type 2 diabetes: a prospective cohort study. BMC Med. 2021;19(1):66. 33715620 10.1186/s12916-021-01936-3 PMC7962330

[18] Zhang Z, Zhao L, Lu Y, et al. Insulin resistance assessed by estimated glucose disposal rate and risk of incident cardiovascular diseases among individuals without diabetes: findings from a nationwide, population based, prospective cohort study. Cardiovasc Diabetol. 2024;23(1):194. doi: 10.1186/s12933-024-02256-5. 38844981 PMC11157942

[19] Si Y, Lee S, Heeringa SG. Population weighting in statistical analysis. JAMA Intern Med. 2024;184(1):98–99. doi: 10.1001/jamainternmed.2023.6300. 38010717

[20] Di Pino A, DeFronzo RA. Insulin resistance and atherosclerosis: implications for insulin-sensitizing agents. Endocr Rev. 2019;40(6):1447–1467. doi: 10.1210/er.2018-00141. 31050706 PMC7445419

[21] Jurczewska J, Ostrowska J, ChełChowska M, et al. Abdominal obesity in women with polycystic ovary syndrome and its relationship with diet, physical activity and insulin resistance: A pilot study. Nutrients. 2023;15(16):3652. doi: 10.3390/nu15163652. 37630842 PMC10459970

[22] Sebastian SA, Padda I, Johal G. Cardiovascular-Kidney-Metabolic (CKM) syndrome: A state-of-the-art review. Curr Probl Cardiol. 2024;49(2):102344. doi: 10.1016/j.cpcardiol.2023.102344. 38103820

[23] Tian C, Liu J, Ma M, et al. Association between surrogate marker of insulin resistance and bone mineral density in US adults without diabetes. Arch Osteoporos. 2024;19(1):42. doi: 10.1007/s11657-024-01395-2. 38796579

[24] Wang T, Lu J, Shi L, et al. Association of insulin resistance and β-cell dysfunction with incident diabetes among adults in China: a nationwide, population-based, prospective cohort study. Lancet Diabetes Endocrinol. 2020;8(2):115–124. doi: 10.1016/S2213-8587(19)30425-5. 31879247

[25] Weinberg AE, Patel CJ, Chertow GM, et al. Diabetic severity and risk of kidney stone disease. Eur Urol. 2014;65(1):242–247. doi: 10.1016/j.eururo.2013.03.026. 23523538 PMC3866968

[26] Ando R, Suzuki S, Nagaya T, et al. Impact of insulin resistance, insulin and adiponectin on kidney stones in the Japanese population. Int J Urol. 2011;18(2):131–138. doi: 10.1111/j.1442-2042.2010.02690.x. 21175865

[27] Kabeya Y, Kato K, Tomita M, et al. Associations of insulin resistance and glycemic control with the risk of kidney stones. Intern Med. 2012;51(7):699–705. doi: 10.2169/internalmedicine.51.6426. 22466823

[28] Kelly C, Geraghty RM, Somani BK. Nephrolithiasis in the obese patient. Curr Urol Rep. 2019;20(7):36. doi: 10.1007/s11934-019-0898-0. 31104149

[29] Qin Z, Zhao J, Geng J, et al. Higher triglyceride-glucose index is associated with increased likelihood of kidney stones. Front Endocrinol (Lausanne). 2021;12:774567. doi: 10.3389/fendo.2021.774567. 34912299 PMC8667164

[30] Carbone A, Al Salhi Y, Tasca A, et al. Obesity and kidney stone disease: a systematic review. Minerva Urol Nefrol. 2018;70(4):393–400. doi: 10.23736/S0393-2249.18.03113-2. 29856171

[31] van der Aa MP, Elst MA, van de Garde EM, et al. Long-term treatment with metformin in obese, insulin-resistant adolescents: results of a randomized double-blinded placebo-controlled trial. Nutr Diabetes. 2016;6(8):e228–e228. doi: 10.1038/nutd.2016.37. 27571249 PMC5022149

[32] Chen D, Xie Y, Luo Q, et al. Association between weight-adjusted waist index and kidney stones: a propensity score matching study. Front Endocrinol (Lausanne). 2024;15:1481393. doi: 10.3389/fendo.2024.1481393. 39286275 PMC11403113

[33] Garofolo M, Gualdani E, Scarale MG, et al. Insulin resistance and risk of major vascular events and all-cause mortality in type 1 diabetes: a 10-year follow-up study. Diabetes Care. 2020;43(10):e139–e141. doi: 10.2337/dc20-0433. 32796028 PMC7510021

[34] Zabala A, Darsalia V, Lind M, et al. Estimated glucose disposal rate and risk of stroke and mortality in type 2 diabetes: a nationwide cohort study. Cardiovasc Diabetol. 2021;20(1):202. doi: 10.1186/s12933-021-01394-4. 34615525 PMC8495918

[35] Ren X, Jiang M, Han L, et al. Estimated glucose disposal rate and risk of cardiovascular disease: evidence from the China Health and Retirement Longitudinal Study. BMC Geriatr. 2022;22(1):968. doi: 10.1186/s12877-022-03689-x. 36517754 PMC9753298

[36] Abate N, Chandalia M, Cabo-Chan AV, Jr., et al. The metabolic syndrome and uric acid nephrolithiasis: novel features of renal manifestation of insulin resistance. K. 2004;65(2):386–392. Kidney Int doi: 10.1111/j.1523-1755.2004.00386.x. 14717908

[37] Iba A, Kohjimoto Y, Mori T, et al. Insulin resistance increases the risk of urinary stone formation in a rat model of metabolic syndrome. BJU Int. 2010;106(10):1550–1554. doi: 10.1111/j.1464-410X.2010.09216.x. 20184575

[38] Spatola L, Ferraro PM, Gambaro G, et al. Metabolic syndrome and uric acid nephrolithiasis: insulin resistance in focus. Metabolism. 2018;83:225–233. doi: 10.1016/j.metabol.2018.02.008. 29510180

[39] Cupisti A, Meola M, D’Alessandro C, et al. Insulin resistance and low urinary citrate excretion in calcium stone formers. Biomed Pharmacother. 2007;61(1):86–90. doi: 10.1016/j.biopha.2006.09.012. 17184967

[40] Khan SR. Reactive oxygen species, inflammation and calcium oxalate nephrolithiasis. Transl Androl Urol. 2014;3(3):256–276. doi: 10.3978/j.issn.2223-4683.2014.06.04. 25383321 PMC4220551

[41] Yoon V, Adams-Huet B, Sakhaee K, et al. Hyperinsulinemia and urinary calcium excretion in calcium stone formers with idiopathic hypercalciuria. J Clin Endocrinol Metab. 2013;98(6):2589–2594. doi: 10.1210/jc.2013-1301. 23553859 PMC3667254

[42] Gillett MJ. International Expert Committee report on the role of the A1c assay in the diagnosis of diabetes: diabetes Care 2009; 32(7): 1327-1334. Clin Biochem Rev. 2009;30(4):197–200. 20011212 PMC2791773

[43] Rossi M, Barone B, Di Domenico D, et al. Correlation between ion composition of oligomineral water and calcium oxalate crystal formation. In: Crystals. 2021;11(12):1507. doi: 10.3390/cryst11121507.

