# Supplementary material for: Association between estimated glucose disposal rate and nephrolithiasis: a propensity score matching study in US adults: results from the National Health and Nutrition Examination Survey 2011–2020
Source: Ren Fail. 2026 Jul 28;48(1):2684346. doi: 10.1080/0886022X.2026.2684346 (PMC13421114; doi:10.1080/0886022X.2026.2684346)
Supplement: Supplemental Material [file IRNF_A_2684346_SM6627.docx]

Table S1 missing data of Variables before multiple imputation

| **Variable** | **Total(n=31,566)** | **Miss(n%)** |
| --- | --- | --- |
| **Sex** |  | 0(0%) |
| **Age** |  | 0(0%) |
| **BMI** |  | 21,016(6%) |
| **Waist** |  | 3,441(10.8%) |
| **Systolic blood pressure** |  | 3,765(11.9%) |
| **Diastolic blood pressure** |  | 3,765(11.9%) |
| **Hemoglobin** |  | 3,050(9.6%) |
| **Creatinine** |  | 3,646(11.5%) |
| **Uric acid** |  | 3,655(11.5%) |
| **Fasting blood glucose** |  | 17,764(56.2%) |
| **HS-CRP** |  | 13,844(43.8%) |
| **Calcium** |  | 3,679(11.6%) |
| **Total cholesterol** |  | 3,541(11.2%) |
| **HbA1c** |  | 3,104(9.8%) |
| **LDL** |  | 18,288(57.8%) |
| **HDL** |  | 3,541(11.2%) |
| **Triglyceride** |  | 18,123(57.3%) |

Table S1 continued

| **Variable** | **Total(n=31,566)** | **Miss(n%)** |
| --- | --- | --- |
| **Drinks** |  | 12,879(40.7%) |
| **High blood pressure** |  | 0(0%) |
| **Race** |  | 0(0%) |
| **Education** |  | 0(0%) |
| **Marital status** |  | 0(0%) |
| **Diabetes mellitus** |  | 0(0%) |
| **Kidney stone** |  | 1(0%) |
| **Weak kidney** |  | 1(0%) |
| **Physical activity** |  | 0(0%) |
| **Smoking** |  | 18,246(57.7%) |
| **PIR** |  | 3,713(11.7%) |

BMI Body mass index, eGDR Estimated glucose disposal rate, HbA1c Glycosylated hemoglobin A1c, HDL High-density lipoprotein, HS-CRP High-sensitivity c-reactive protein, LDL Low-density lipoprotein,

PIR Household income–to–poverty ratio.
