# Supplementary material for: Association between estimated glucose disposal rate and nephrolithiasis: a propensity score matching study in US adults: results from the National Health and Nutrition Examination Survey 2011–2020
Source: Ren Fail. 2026 Jul 28;48(1):2684346. doi: 10.1080/0886022X.2026.2684346 (PMC13421114; doi:10.1080/0886022X.2026.2684346)
Supplement: Supplemental Material [file IRNF_A_2684346_SM6622.docx]

Table S3 Weighted multivariable logistic regression for the association between the varieties and nephrolithiasis before Propensity Score Matching

|  | **OR (95%CI), P-value** |
| --- | --- |
|  | **Basic model ^a^**  +hypertension  + HbA1c  + Waist |
| **eGDR** | 0.90 (0.88, 0.93)  <0.001 |
|  | **Basic model**  +eGDR |
| **High blood pressure** |  |
| No | Reference |
| Yes | 1.07 (0.89, 1.30)  0.5 |

**^a^** Basic Model adjusted the cofactors including age, gender, race, physical activity, education level, marital status, PIR, smoking, drink, diabetes mellitus, total cholesterol, triglyceride, LDL, HDL, HS-CRP, fasting blood glucose, hemoglobin, weak kidney, calcium, and creatinine.
